# Environmental epigenetic modifiers in neurodegenerative diseases: a systematic review of epigenomic mechanisms and therapeutic targets

**DOI:** 10.3389/freae.2026.1833533

**Published:** 2026-05-28

**Authors:** Pranay Wal, Sunita Mishra, Debashish Pradhan, Ankita Wal, Debashis Purohit, Lalit Mohan Nainwal, Vivek Dhir, Amin Gasmi

**Affiliations:** 1 Pranveer Singh Institute of Technology (Pharmacy), Kanpur, India; 2 Department of Pharmaceutical Chemistry, College of Pharmaceutical Science, Puri, Odisha, India; 3 Department of Pharmacology, Utkal University, Bhubaneswar, Odisha, India; 4 MATS School of Pharmacy, Mats University, Raipur, Chattisgarh, India; 5 Teerthanker Mahaveer College of Pharmacy, Teerthanker Mahaveer University, Moradabad, Uttar Pradesh, India; 6 Department of Pharmacology, Punjab Multipurpose Medical Institute, Barnala, Punjab, India; 7 International Institute of Nutrition and Micronutrition Sciences, Saint-Étienne, France; 8 Société Francophone de Nutrithérapie et de Nutrigénétique Appliquée, Villeurbanne, France

**Keywords:** environmental neurotoxicants, epigenetics, DNA methylation, histone modification, non-coding RNA, neurodegenerative diseases, particulate matter

## Abstract

**Background:**

Environmental contaminants and neurotoxicants are becoming more widely acknowledged as major causes of neurodegenerative illnesses due to systemic biological disruption including oxidative stress, mitochondrial dysfunction, chronic neuroinflammation, and chromatin remodeling–associated epigenetic dysregulation epigenetic modification. According to new research, exposures to heavy metals, gaseous pollutants, particulate matter, and synthetic neurotoxicants alter gene-regulatory networks and accelerate neurodegenerative processes.

**Objective:**

With an emphasis on molecular mechanisms, developmental neurotoxicity, and translational pathways connecting environmental exposures with epigenomic changes and neurological outcomes, the goal of this systematic review is to compile the most recent data on environmental epigenetic modifiers in neurodegenerative diseases.

**Method:**

Using structured searches across major biomedical databases, the review was carried out in accordance with PRISMA principles. A multistage selection procedure was used to evaluate studies that addressed environmental neurotoxicants, epigenetic processes (DNA methylation, histone modification, and non-coding RNAs), and neurodegenerative consequences. To find common routes and life-course exposure effects, relevant experimental, epidemiological, mechanistic, and translational data were qualitatively summarized.

**Results:**

The findings of the included research show that exposure to the environment causes oxidative stress, mitochondrial dysfunction, neuroinflammation, and chromatin remodeling. These effects result in alterations in histone acetylation, DNA methylation patterns, and dysregulated expression of microRNAs. Amyloidogenic signaling, synaptic dysfunction, and neuronal death were consistently linked to both chemical and particulate matter neurotoxicants. Furthermore, early environmental stressors create long-term epigenetic programming that heightens vulnerability to eventual neurodegenerative pathology, according to developmental exposure models.

**Conclusion:**

An important link between the exposome and the risk of neurodegenerative diseases is represented by environmental epigenetic modifiers. Epigenetic pathways including histone acetylation imbalance, microRNA-mediated regulation (e.g., miR-574-5p), and DNA methylation changes emerge as potential biomarkers and therapeutic targets. Future research should prioritizemulti-omics techniques, harmonized exposure assessment, and longitudinal human investigations to advance precision prevention and intervention strategies.

## 1 Introduction

Parkinson’s disease (PD), Alzheimer’s disease (AD), along with other dementias are examples of neurological disorders that are becoming more widely acknowledged as serious public health issues with significant effects on people, families, and societies around the world (Scheltens et al., 2021). The worldwide shift in demographics, which is marked by an aging population, is intimately linked to the expanding incidence of these disorders. The need to treat a growing epidemic of neurodegenerative illnesses has become more urgent as a result of this demographic transition and the possible long-term repercussions of the COVID-19 pandemic vascular problems, post-viral immunological dysregulation, persisting neuroinflammatory reactions, and the documented amplification of cognitive decline and Parkinsonian symptoms in post-infectious cohorts. Recent revisions to the Global Burden of Disease (GBD) 2021 research (Ben-Shlomo et al., 2024) and the GBD 2021 assessment for neurological Disorders offer the most recent data to inform advocacy and future efforts (Steinmetz et al., 2024). Importantly, over 40% of the world’s population was impacted by neurological illnesses, which caused an 18.2% (95% UI 8.7–26.7) rise in DALY counts worldwide between 1990 and 2021 (Wang S. et al., 2024). Global population structures are aging, which raises the prevalence and burden of neurodegenerative illnesses and is one of the main causes of a rising number of brain diseases. Millions of people are impacted by these illnesses, which account for a sizeable amount of the world’s disease burden and have high medical, social, and financial implications (Iwarsson et al., 2023).

Beyond just their patients, neurodegenerative diseases (NDD) also have an influence on their caregivers, who frequently experience material, physical, and emotional hardships (Wells et al., 2021). Furthermore, there is growing pressure on medical care in various nations and territories to offer older adults with NDD appropriate, accessible, and efficient care; however, in many cases, this is still not the case due to a shortage of funding, specialized expertise, and feasible policy systems and approaches. A thorough analysis that outlines the present situation and expected future development of neurodegenerative illnesses, pinpoints the gaps in current care models, and suggests ways for advancement is desperately needed in light of these difficulties (Geerlings et al., 2023).

In addition to aging, the most prevalent risk factors for neurodegenerative illnesses are smoking and alcohol consumption, gender, and a medical record of diabetes, hypertension, cranial damage, tumors, and infections (Pang et al., 2019). On the other hand, little is known about how the majority of these diseases establish, particularly the potential role of environmental factors. Since 91% of the global populace lives in areas where air pollution levels are higher than those set by the World Health Organization (WHO), this review concentrates on air quality because of its substantial global impact (Oliveira et al., 2024).

According to recent research, air pollution, particularly particulate matter with a diameter of less than 2.5 µm (PM2.5), can activate the mechanisms that cause neurodegenerative disorders because it can enter the brain (Parra et al., 2022). Air pollution can enter the brain through four main pathways: nasal olfactory (the inhalation of ultrafine and tiny fragments that enter the lungs. Further they enter the circulatory system, including the trigeminal nerve, the spinal cord, the hippocampus, and the frontal lobe); systemic irritation (in and this the pollutant damages peripheral organs like the lungs, causing a broader reaction that propagates the inflammation to the brain) (Murata et al., 2022); and particulate matter serves as a transporter for metals, organic compounds, and reactive chemicals that separate from particle surfaces and pass through biological membranes regardless of particle size in adsorbed toxicant transport processes (Roy and D’Angiulli, 2024). Inhaled oxidation occurs when reactive oxygen species, such ozone, communicate with lipids and proteins in the respiratory tract, changing them into harmful substances that cause oxidative stress and cause different types of brain injury (Flood-Garibay et al., 2023), Figure 1. AD, PD, dementia, autism, and stroke are all linked to PM2.5 (Chauhan et al., 2025; P. Fu and Yung, 2021). The central nervous system (CNS) is also poisoned by other pollutants like ozone (O3), particulate matter having a diameter of less than 10 µm (PM10), nitrogen dioxide (NO2), carbon monoxide (CO), and sulfur dioxide (SO2) (Shah et al., 2015). Although there are many research on neurodegenerative illnesses and the impact of the natural world on the CNS, there are few that use a geospatial strategy, and most of them just take the geographic area into account as a clustering element, without going beyond (Oliveira et al., 2024).

**FIGURE 1:**
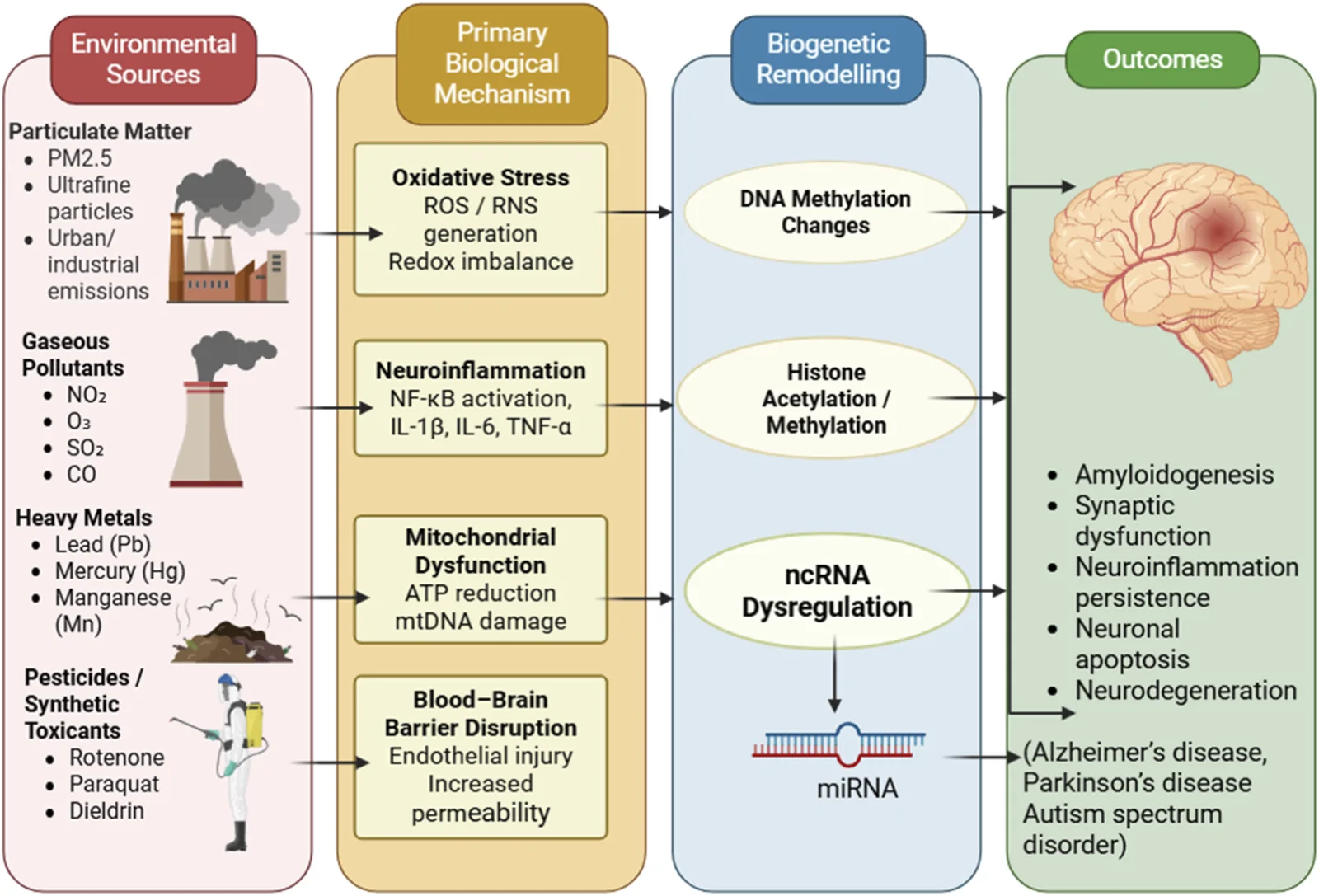
Environmental exposome–epigenome framework linking neurotoxicant exposure to neurodegenerative disease risk.

The expression of genes related to stress responses, neurological protection, and synaptic plasticity may be impacted by these epigenetic changes. Early identification and surveillance of CNS diseases may be facilitated by knowledge of the connection between neurological disorders and epigenetic modifications brought on by particular air contaminants. To far, however, attempts have been made provide how NDD risk influences cellular phenotypes from genetics, aging, or environmental exposures. It may be possible to identify the molecular mechanisms underlying the start and advancement of NDD by combining known relationships between these variables with gene regulatory and epigenetic markers (Tsalenchuk et al., 2023). Investigating the epigenetic mechanisms implicated in NDD has gained more attention recently, primarily due to the possibility that these pathways connect genetic information with environmental stressors. The majority of risk loci and more than 90% of single nucleotide polymorphisms (SNPs) are found in non-coding areas of the genome, most likely through modifying the activity of gene regulatory elements such as enhancers and promoters. It has been helpful to understand genetic risk in NDD by using gene regulatory tagging to comprehend noncoding risk elements from GWAS. This has shown, for instance, which cell types are most impacted by genetic risk variants (Bryois et al., 2020).

### 1.1 Objective

The purpose of this systematic review is to assess environmental neurotoxicants’ function as epigenetic modifiers in neurodegenerative disorders including neurodevelopmental diseases linked to autism, PD, AD, dementia spectrum disorders, and associated neurodegenerative phenotypes impacted by environmental exposure. Its main goal is to comprehend how contaminants like metals, gaseous neurotoxicants, and particulate matter affect DNA methylation, histone modification, and the control of non-coding RNA.

Using a PEO framework, the review question was organized as follows:

Population (P): Human and experimental models of neurodegenerative disease

Exposure (E): Particulate particles, heavy metals, gaseous pollutants, and synthetic chemicals are examples of environmental neurotoxicants.

Outcome (O): Neurological consequences linked to epigenetic changes (DNA methylation, histone modification, non-coding RNA regulation)

Understanding how pollutants such as metals, gaseous toxicants, and particulate matter impact DNA methylation, histone modification, and the regulation of non-coding RNA is its primary objective.

## 2 Methodology

### 2.1 Study design and protocol framework

In order to compile the most recent data on environmental neurotoxicants, epigenetic processes, and the consequences of neurodegenerative diseases, this work was carried out as a systematic review. To guarantee openness, reproducibility, and organized reporting, the review design adhered to the Preferred Reporting Items for Systematic Reviews and Meta-Analyses (PRISMA) framework. The review’s objectives were to find, compile, and analyze data about environmental neurotoxicants and their epigenetic impact on neurodegenerative illnesses. This review was not listed in any public systematic review registry, including PROSPERO.

### 2.2 Literature search strategy and databases

A structured literature search was performed across multidisciplinary biomedical and environmental science databases. The strategy targeted peer-reviewed articles addressing environmental pollutants, developmental neurotoxicity, epigenetic alterations, and neurodegeneration. Keywords and thematic combinations were derived from core manuscript concepts, including:

“environmental neurotoxicants,”

“air pollution,” “particulate matter,” “heavy metals,”

“epigenetics,” “DNA methylation,” “histone modification,” “microRNA,”

“neurodegenerative diseases,” “developmental neurotoxicity,” and “oxidative stress.”

Database queries were limited using Boolean operators and structured search strings, such as: (“environmental neurotoxicants” OR “air pollution” OR “particulate matter”) AND (“epigenetics” OR “DNA methylation” OR “histone modification” OR “microRNA”) AND (“neurodegenerative diseases” OR Alzheimer’s disease” OR Parkinson’s disease”. To maximize sensitivity without sacrificing relevance, database-specific filters and truncation symbols were also used when applicable. Both experimental and human-based studies were considered to capture mechanistic, translational, and epidemiological perspectives discussed in the manuscript’s developmental neurotoxicity and epigenetic sections. Reference lists of relevant articles were also screened to identify additional eligible studies.

### 2.3 Inclusion and exclusion criteria

Studies were included if they:

- Studied environmental pollutants or neurotoxicants (e.g., particulate matter, gaseous pollutants, pesticides, metals, synthetic toxins).
- Potential epigenetic processes such as DNA methylation modification, histone modification, and non-coding RNA change were documented.
- Studied neurodevelopmental, neurodegenerative or neurological effects.
- Only primary research studies—experimental *in vivo*, *in vitro*, and human epidemiological studies—that provided unique information pertinent to environmental exposure and epigenetic mechanisms were included.
- Review articles (narrative or systematic) were excluded from quantitative and qualitative synthesis but were used only for background context where necessary.
- Published between January 2016 and January 2026.

Studies were excluded if they:

- Were limited to genetic alterations only with no explanation of environmental exposure.
- Lacked mechanistic or epigenetic relevance.
- Did not have associations with neurodevelopmental or neurodegenerative processes.

### 2.4 Screening and study selection process

In accordance with PRISMA guidelines, a multi-stage screening procedure was applied to all identified records. First, titles and abstracts were assessed for their applicability to neurodegeneration and environmental epigenetics. Studies that might be qualified moved on to full-text evaluation. Studies were assessed for conformity to the manuscript’s thematic focus on developmental neurotoxicity, systemic processes, translational models, and epigenetic modifications *via* full-text screening. To guarantee conformity with the established review objectives, disagreements about eligibility were settled by consensus.

### 2.5 Data extraction and management

Data were extracted using a standardized framework designed around the manuscript’s analytical structure. Key information collected from each study included:

- Type of environmental exposure and source.
- Experimental model or population characteristics.
- Molecular pathways and cellular mechanisms.
- Epigenetic outcomes (DNA methylation, histone changes, ncRNA regulation).
- Neurological or neurodegenerative endpoints.

Extracted data were organized into comparative tables reflecting environmental neurotoxicants, mechanistic pathways, and translational models, as presented in the manuscript’s tabulated summaries.

### 2.6 Quality assessment and risk of bias evaluation

Methodological rigor, exposure characterisation, and translational relevance were the main focuses of the quality assessment. Special attention was paid to the constraints mentioned in the study, such as the heterogeneity of epigenetic measuring platforms, the dependence on animal models or *in vitro* systems, and the variability in exposure assessment.

Risk of bias considerations included:

- Inconsistencies in pollutant composition or exposure duration.
- Differences between experimental and human study designs.
- Short-term observational epigenetic findings without longitudinal confirmation.

These considerations guided interpretation rather than quantitative scoring, consistent with the narrative-mechanistic nature of the review.

### 2.7 Data synthesis and analytical strategy

A systematic qualitative synthesis approach was used because exposure models, molecular outcomes, and study designs varied.

Exposure type, biological model, and epigenetic outcome variables were used to comprehensively classify the studies.

To provide uniformity in interpretation, mechanistic discoveries and epidemiological data were combined using pre-established theme groups that were in line with the PEO framework.

Evidence was grouped according to thematic domains:

- Developmental neurotoxicants and particulate matter exposure.
- Environmental neurotoxicants as epigenetic modifiers.
- Translational models and systemic pathways.
- Epigenetic mechanisms including DNA methylation, histone modification, and non-coding RNAs.

Mechanistic findings and epidemiological facts were integrated through cross-sectional comparisons, highlighting the life-course susceptibility framework and exposome-based model discussed in the text.

## 3 Results

### 3.1 Study selection and PRISMA flow diagram

A systematic literature search was conducted following the PRISMA guidelines. A total of 360 records were identified from PubMed (n = 188), Web of Science (n = 77), and Google Scholar (n = 95). Before screening, 74 records were removed, including duplicates (n = 31) and articles excluded for other reasons such as reviews, editorials, or irrelevant scope (n = 43). The remaining 286 records underwent title and abstract screening, during which 60 studies were excluded due to lack of environmental/epigenetic focus or absence of relevant mechanistic outcomes. Subsequently, 226 reports were sought for full-text retrieval, of which 31 were not accessible, leaving 195 articles assessed for eligibility. After full-text evaluation, 71 reports were excluded because they lacked experimental environmental neurotoxicant components (n = 19), were not directly related to neurodegenerative epigenetic mechanisms (n = 27), had insufficient methodological detail (n = 9), or contained overlapping datasets (n = 16), Figure 2. Finally, 124 studies met the inclusion criteria and were incorporated into the qualitative synthesis.

**FIGURE 2:**
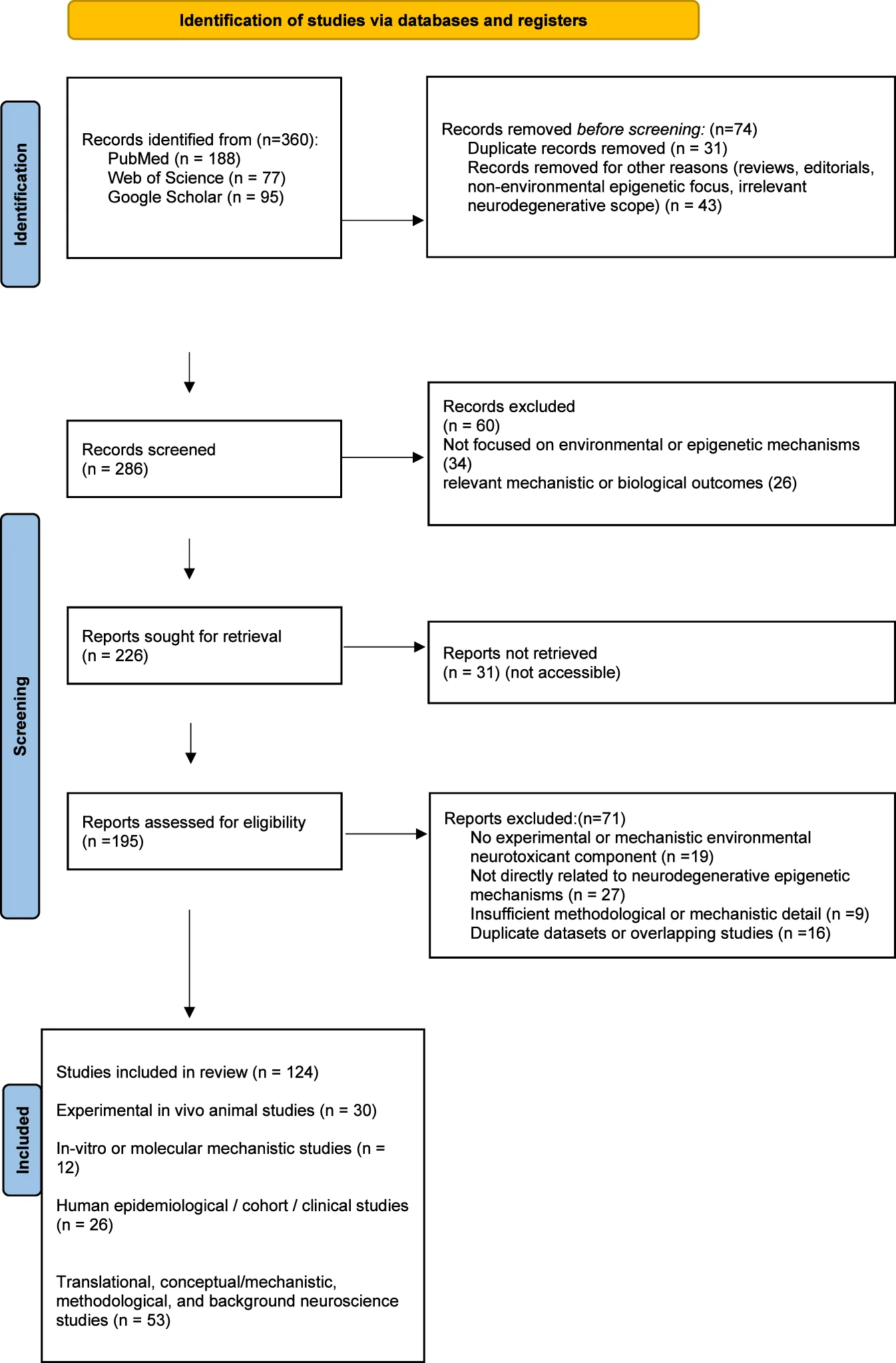
PRISMA flow diagram.

### 3.2 Characteristics of included studies

A comprehensive body of research connecting environmental neurotoxicants to epigenetic remodeling and neurodegenerative diseases was represented by the selected papers. *In-vitro* mechanistic cellular studies, human epidemiological cohort analyses, case-control investigations, experimental *in vivo* animal studies reports were among the included literature. Sample sizes ranged from large population-based cohorts with hundreds to thousands of participants to controlled cellular experiments. Reactive oxygen species production, NF-κB activation, mitochondrial damage, and chromatin remodeling processes that affected DNA methylation patterns, histone acetylation levels, and microRNA expression profiles were among the common molecular themes that surfaced across investigations. Together, these results validated the integrated model put forth in the study, according to which environmental exposures alter gene-regulatory networks and increase the risk of neurodegenerative diseases throughout life.

### 3.3 Developmental neurotoxicants and particulate matter

Neurotoxicants, also referred to as developmental neurotoxicity (DNT), are chemical compounds that are known to cause adverse impacts on nervous systems after exposure during the development of the CNS throughout the life cycle (D’Angiulli, 2019). These materials can include organic solvents, medicines, and chemicals used in industry. Because they require a dynamic convolution to approximate the degree of exposure and determine their “dosage” in an environmental context, neurotoxicants are intrinsically difficult to quantify. An estimated 214 neurotoxicants have been documented during 2006 and 2013 (Tywabi-Ngeva et al., 2022), and new estimates indicate that almost 30,000 compounds, or about 30% of substances employed in commerce, might possess neurotoxic capacity (Legradi et al., 2018). The variety of recognized neurotoxicants is increasing annually. Since experimental research in laboratories show that approximately 1,000 compounds are neurotoxic to animals, suggesting several may potentially be dangerous to people, scientists are continuously keeping an eye on this number (Liu T. et al., 2026). Additionally, over 300 chemicals included in food have been linked to adverse consequences on brain growth; the greatest category of official neurotoxicants is pesticides (Botnaru et al., 2025).

Aiming to enhance the determination of neurotoxic effects by means of multilevel investigation, including clinical, personality, and neuropathological examinations, the United States Environmental Protection Agency (USEPA) and the Organization for Economic Co-operation and Development (OECD) developed standards (Fouyet et al., 2024). Numerous studies show that neurotoxicants disrupt biological functions like cell migration and proliferation, with infants’ and children’s maturing nervous systems being especially susceptible. The complex mixes of liquid and solid particles released by automobiles, factories, and home heat pumps make up particulate matter (PM), a significant environmental neurotoxicant. While ultrafine particulate matter (UFPM) has an aerodynamic radius below 100 nm, PM10 contains particles smaller than 10 µm. Due to their ability to penetrate biological barriers including the blood–brain barrier and their ability to stay in the air, PM and UFPM can both have neurotoxic effects (Armas and D’Angiulli, 2022). The World Health Organization estimates that in 2010, ambient air pollution caused around 3.1 million fatalities and exposure to PM2.5 had been linked to an estimated 8.6-month decrease in life expectancy. These particles cause oxidative stress, ROS generation, and inflammatory reactions, all of which support neurological disorders and structural changes in the CNS (Yin et al., 2022).

Animal studies have demonstrated that oxidative stress causes neurodegeneration, while chemicals such ROS can induce tissue damage after prolonged exposure. Many studies look at the potential function of neurotoxicants in causing neuroinflammation, especially the biomolecular changes that follow exposure (Arias-Pérez et al., 2020). Among the most widely used methods is the laboratory exposure of cells to PM, and research indicates that inflammation is an underlying trait that sets various diseases linked to air pollutants apart. The length and severity of exposure are two interrelated parameters that determine how much biological change is caused by neurotoxicants (Fu C. et al., 2022). Three categories are used to structure assessing exposure inside the exposome idea: internal biological variables, particular external exposures, and broad external environments. Although PM has been extensively described in the literature as a test paradigm for developmental neurotoxicity, little is known about numerous other environmental substances. Commonly found pollutants that can exacerbate oxidative stress, interfere with neurotransmitter release, and cause neurodevelopmental disorders include heavy metals such arsenic, cadmium, lead, and mercury (Pandics et al., 2023).

Several research provide narrative descriptions of mechanistic processes inside the text in addition to tabulated evidence. Table 1 presents an integrated perspective of exposure–mechanism correlations by synthesizing these narrative mechanistic findings.

**TABLE 1:** Comprehensive overview for studies showing narratively explained mechanistic pathways connecting environmental contaminants to neurological consequences (Except for tabulated data, only studies with mechanisms specifically mentioned in the text are included).

Study (author, year)	Exposure	Model/Context	Mechanistic pathway (narrative only)	Key neurobiological outcome
Yin et al. (2022)	Particulate matter (PM, UFPM)	General environmental exposure	Induces oxidative stress, ROS generation, and inflammatory responses; capable of crossing BBB and accumulating in CNS	Structural CNS changes, neurodegeneration
Fu et al. (2022)	Neurotoxicants	Exposome framework	Biological impact depends on exposure duration and intensity, influencing cumulative molecular damage	Dose-dependent neurotoxicity
Pandics et al. (2023)	Heavy metals (As, Cd, Pb, Hg)	Environmental exposure	Promote oxidative stress, neurotransmitter disruption, and interference with neuronal signaling	Neurodevelopmental disorders
Jin et al. (2024)	PM exposure (prenatal/postnatal)	Human developmental	Induces brain injury during development via inflammation and oxidative stress pathways	ASD and neurodevelopmental risk
Hameed et al. (2020)	UFPM, cytokines	Circulatory pathway	Causes endothelial damage, BBB disruption, Aβ deposition, and neurofibrillary tangles	Neurodegeneration (AD-like)
Li et al. (2018)	PM2.5	Molecular pathway	Activates NFκB → BACE1 upregulation → Aβ production; miR-574-5p acts as protective regulator	Amyloidogenesis, cognitive decline
Yang et al. (2022)	Gut–brain axis	Neurodegeneration models	α-synuclein propagation from gut to brain via vagal pathways	PD progression
Jiang et al. (2025)	PM2.5	Molecular	Alters DNA methylation (CpG sites) in neuroinflammation genes	Epigenetic instability
Ku et al. (2017)	PM2.5	Molecular	Suppresses miR-574-5p → increases BACE1 → synaptic dysfunction	Cognitive impairment
Stoccoro and Coppedè (2024)	Mixed pollutants	Conceptual synthesis	Oxidative stress + neuroinflammation act upstream of epigenetic dysregulation (DNA methylation, histone changes, ncRNA)	Persistent disease risk

### 3.4 Environmental neurotoxicants as epigenetic modifiers in neurodegenerative diseases

Through mechanisms facilitated by oxidative stress, neuroinflammation, mitochondrial dysfunction, and epigenetic abnormalities, environmental pollutants and chemical neurotoxicants constitute significant external modulators of the course of NDDs. In addition to pesticides, heavy metals, and synthetic neurotoxicants, gaseous pollutants such as ozone, nitrogen oxides (NOx), sulfur dioxide (SO2), and carbon monoxide (CO) can enter the CNS directly using the olfactory system or indirectly by systemic circulation, where they disrupt neuronal homeostasis (Kalenik et al., 2025). These exposures cause DNA methylation, ROS production, altered histone acetylation, miRNA expression, and pathogenic protein aggregation. Environmental neuro-toxicants like MPTP, rotenone, paraquat, dieldrin, manganese, and vanadium may cause inflammation signaling pathways, dysregulate functional mitochondria, and change the patterns of gene expression of genes in important counterbalancing clusters linked to the decision-making process for neuronal survival, according to experimental and epidemiological data (Huang et al., 2022). These environmental neurotoxins act as molecular frameworks that link neurodegeneration to exposure to epigenetic changes in the genome.

An overview of the most common gaseous pollutants and neurotoxicants, along with information on their mode of action, epigenetic modifications, and documented neurological findings, is provided in Table 2.

**TABLE 2:** Neurotoxicants and environmental contaminants linked to the pathophysiology of neurodegenerative diseases and epigenetic modification.

Category	Agent	Major exposure source	Molecular mechanisms	Epigenetic/cellular effects	Neurodegenerative outcomes	Evidence type	References
Gaseous pollutant	**Ozone (O3)**	Photochemical reactions of NOx and VOCs	ROS generation, cytokine activation, microglial dysfunction	Reduced neurogenesis, **dendritic spine loss**	AD-like pathology, PD risk	Animal + Cohort	Ramírez-Mendoza et al., 2024; Olloquequi et al., 2024
	**Sulfur Dioxide (SO2)**	Fossil fuel combustion, industrial emissions	COX-2 signaling, **inflammatory cytokines**	**Synaptic damage,** Arc gene suppression	Cognitive decline, MS, AD progression	Animal + Epidemiology	Dhapola et al. (2024)
	**Nitrogen Oxides (NOx/NO2)**	Combustion, traffic emissions	**Excitotoxicity**, prostaglandin signaling	**Amyloid accumulation,** synaptic plasticity impairment	Dementia, ASD, PD	Cohort + Animal	Yang Y. et al., 2025; Li H. et al., 2024
	**Carbon Monoxide (CO)**	Incomplete combustion	Hypoxia, mitochondrial dysfunction	Nitrosative stress, demyelination	Parkinsonism, cognitive deficits	Clinical + Toxicology	Afzal et al. (2025)
Neurotoxicant	**MPTP**	Synthetic toxin exposure	Complex I inhibition, **ROS**	**α-synuclein aggregation,** apoptosis	PD models	Experimental	Qu et al. (2019)
	**Rotenone**	Botanical pesticide	Mitochondrial inhibition, microtubule destabilization	Autophagy dysfunction	PD pathology	Epidemiology + Animal	Ibarra-Gutiérrez et al. (2023)
	**Paraquat**	Herbicide exposure	ROS generation	Histone acetylation dysregulation	PD risk	Experimental	Sharma and Mittal, (2024)
	**DDT**	Organochlorine pesticide	Membrane depolarization, mitochondrial dysfunction	**Oxidative stress signaling**	AD biomarker association	Experimental	Farkhondeh et al. (2020)
	**Dieldrin**	Persistent insecticide	Proteasomal dysfunction, **oxidative stress**	**Histone hyperacetylation,** DNA methylation	Dopaminergic degeneration	Epidemiology + *In vitro*	Stoccoro and Coppedè, 2024; Yu et al., 2021a
Metal	**Manganese**	Industrial dust, mining	**Neuroinflammation, ROS, mitochondrial dysfunction**	**DNA methylation, miRNA alteration**	Manganism, PD-like symptoms	Human + Animal	Monti et al., 2025; Pathak and Krishnan, 2023
	**Vanadium**	Fossil fuel combustion, industry	MAPK signaling, **oxidative stress**	Caspase activation, inflammatory pathways	Motor and memory deficits	Experimental	Adekomi et al. (2025)

Major neurodegenerative mechanisms highlighted in bold for emphasis.

## 4 Particulate matter induced brain injury in humans

Since 1992, Calderón-Garcidueñas stated findings on the impacts of severely polluted air in large towns, wherein the total load of pollutants was linked to a range of changes in several human and canine organs and tissue types (Nunez et al., 2022). But it wasn’t till 2015 that PM2.5 was linked to certain disease markers for conditions like obesity, AD, PD, ALS, non-Alzheimer’s dementia (N-AD), and others (Mousavi et al., 2017). Additionally, it has been hypothesized that oxidative stress and inflammation disrupt the blood-brain barrier, resulting in persistent neuroinflammation, and that PM10, SO2, NO2, and NO have been developed linked to deteriorating outcomes for MS patients. After these occurrences, there may be an immunological assault bolstered by transcriptional factors including activated microglia (microgliosis), which targets neuronal tissue and significantly reduces self-tolerance and may result in the generation of autoantibodies (Roux et al., 2017). Additionally, prenatal and postnatal brain injury has been linked to PM air pollution, namely, the observed rise in autism and autism spectrum disorder (ASD) (Jin et al., 2024).

Delays in the progression of brain maturation pathways has been linked to prenatal exposure to PM in heavily polluted urban areas. Neurological disorders like schizophrenia and ASD which undergo clinical diagnosis in the early years may be exacerbated by such retardation. Boys appear to be more vulnerable than girls to this change in brain growth, which is further proven by behavioral changes, cognitive problems, consolidating memories, motor language difficulties, and lower intellectual ability (Klocke et al., 2018). Neurodegenerative disease indicators are found and other alterations take place when PM2.5 exposure rises between 5 months prior to delivery and 1 month postpartum (perinatal). It appears that boys’ brains take longer to mature than girls’, therefore the EADS is less effective, and exposure to PM2.5 and NO2 has been shown to cause significant harm. The negative impacts on verbal and memory retention were substantial. Additionally, NO2 had a major impact on affectation in global cognition, which includes motor and numerical skills (Calderón-Garcidueñas et al., 2021). The most significant impacts might be caused by reduced fetal growth, systemic and neuroinflammation, and oxidative stress. Similar findings have been reported in experimental settings where sex and exposure duration (prenatal and/or postnatal) are also controlled. Impulsivity-like behavior resulted from impairments in memory, cognition, and behavioral flexibility. Despite the constant activation of microglia in both males and females, they were variations in the amino acid supply that were likely associated with sexually distinct neurotoxicity (Cory-Slechta et al., 2023).

Immediate postnatal exposure to PM2.5 raises the risk of attention deficit hyperactivity disorder (ADHD) and autism spectrum disorders (ASD). Because early postnatal exposure to PM2.5 is persistent, it is challenging to distinguish the changes in neural growth from other possible causes (Shang et al., 2023). In addition, the neurodevelopmental pace in animal models (28 days) against the 3 years needed in humans presents significant challenges for the standardization of clinical models. As a result, the order in which the molecules implicated sustain injury and the organisms’ ability to respond may change in both situations. Late childhood (3 years and older) exposure to PM-polluted air has been linked to the development of indicators of depression and anxieties as well as a spike in ADHD (Kim et al., 2021).

## 5 Particulate matter induced neurodegenerative markers

Since both PM2.5 and UFPM have a BCC and can extensively diffuse into the respiratory system, as was previously indicated, the adsorbed molecules can differ based on their relative number and existence in the inhalable air. The olfactory neurons found in the olfactory mucosa serve as these PMs’ initial point of entry into the central nervous system, Table 3. Although the intracellular the existence of UFPM in the gustatory neurons of a rodent model subjected to synthetic BCC has previously been linked to an increased release of glutamate and glycine, along with inflammatory cytokines, after 6 and 11 h of exposure, the integration of UFPM in humans appears to be less than 3.5% (Saveleva et al., 2023).

**TABLE 3:** Biological effects and the mechanistic pathway linked to particulate matter exposure.

S.No	Entry route/Exposure interface	Primary cellular targets	Molecular and signaling events	Neurodegenerative biomarker output	Experimental or human evidence	References
1.	Olfactory mucosa → olfactory bulb neurons	Olfactory neurons, **glial cells**	Glutamate and glycine release; inflammatory cytokines ↑	**Aβ immunoreactivity; α-synuclein accumulation**	Mouse olfactory bulb model; MMC human observations	Zhao et al., 2024; Zahr, 2024
2.	Systemic circulation after alveolar macrophage interaction	Endothelial cells; **BBB** structures	RONS generation; oxidative stress; inflammatory cytokines	**BBB disruption; neurofibrillary tangles; Aβ deposition**	Circulatory diffusion studies	Gu M. et al. (2021)
3.	PM2.5-induced nitrooxidative stress	Neurons expressing APP	NFκB activation; BACE1 overexpression; miR-574-5p modulation	**Increased Aβ generation;** altered synaptogenesis	Experimental PM exposure models	Jiang et al. (2025)
4.	Excitotoxic susceptibility of hippocampal neurons	**Glutamatergic neurons**	Caspase-3 activation; dose-dependent viability decrease	**Neuronal apoptosis signaling**	Cellular neurotoxicity studies	Nikbakhtzadeh et al. (2025)
5.	Microglial activation and oxidative cascade	**Dopaminergic neurons**	O2− generation; RONS amplification	PD risk–related neuronal loss	Neurodegeneration models	Simpson and Oliver, (2020)
6.	Intracellular particle accumulation	Mitochondria and ER contact sites	Permeability transition; ATP decrease; mtDNA copy number ↓	Mitochondrial dysfunction markers	Ultrastructural cellular analyses	Szymański et al. (2017)
7.	BCC-associated oxidant pollutants	**Neurons and microglia**	NFκB activation; inflammatory interleukins and cytokines expression	Sustained neuroinflammatory signaling	Nitrooxidative stress pathway studies	Teng et al. (2021)
8.	Olfactory bulb endothelial changes	Endothelial and vascular cells	Endothelial hyperplasia; inflammatory signaling	**Increased Aβ and α-synuclein immunoreactivity**	MMC neuropathological findings	Bogale et al., 2021; Calderón-Garcidueñas et al., 2018
9.	Systemic inflammatory mediators	Circulating immune cells	Cytokine transport across BBB; oxidative signaling	**Neuroinflammation** and neuronal damage	Peripheral-to-central inflammatory models	Tan et al. (2023)
10.	PM-induced neurotransmitter imbalance	**Synaptic terminals**	Excitotoxic glutamatergic signaling	Synaptic dysfunction	Neurotransmitter release studies	Gui et al. (2023)
11.	Amyloidogenic enzymatic pathway activation	APP-processing enzymes	BACE1 upregulation following NFκB activation	**Amyloid plaque formation**	Molecular signaling studies	Zhang et al., 2019; Krawczuk et al., 2025
12.	Organelle structural alterations	Mitochondria and ER	Mitochondrial dissociation; ER contact alterations	Energy metabolism impairment	Intracellular structural analyses	Stacchiotti et al. (2018)

Major neurodegenerative mechanisms highlighted in bold for emphasis.

*Via* the bloodstream, UFPM, RONS, and inflammatory cytokines enter the brain and cause endothelial cell destruction, oxidative damage, neuroinflammation, Aβ deposition, and neurofibrillary tangles, as well as alter the integrity of the blood-brain barrier (Hameed et al., 2020). Neurological Aβ plaques have been linked to increased PM2.5 exposure for individuals with dementia or mild cognitive dysfunction. Additionally, in adolescents and young adults, the growth of Aβ plaque occurs prior to a neuroinflammatory state. Exposure to PM2.5 causes an overexpression of the beta-site amyloid precursor protein cleaving enzyme (BACE1). BACE1 can be suppressed by miR-574-5p, while its expression is downregulated by NFκB activation. It works on the amyloid precursor protein and produces Aβ. As a result, when PM2.5-induced nitrooxidative stress triggers NFκB and BACE1, Aβ is produced. Thus, following interaction with PM2.5, miR-574-5p has a protective impact on sinaptogenesis and enhances cognition, comprehension, and memory (Li B. et al., 2018).

The primary causes of a vicious loop or harm spiral are neuroinflammation and nitrooxidative damage. Both the BCC core and the adsorption oxidant contaminants that encircle it are strong RONS inducers, that trigger NFκB and cause the production of inflammatory cytokines and interleukins (Singh S. et al., 2023). Abnormal mitochondrial shape, mitochondrial dissociation, and endoplasmic reticulum connections with a buildup of intracellular PM are among the intracellular alterations brought on by PM. Modifications in mitochondria include low mtDNA copy amount, diminished ATP generation, pore widening with permeability transition, and lowered mitochondrial capacity (Mendoza-Magaña et al., 2021).

## 6 Inflammation and apoptosis in neurons

PM, UFPM, PAH, and other organic chemicals are the main topics of the vast volume of publications on air pollution. Unquestionably, the often researched neurodevelopmental toxic chemicals (such neurotoxicants) represent a very minor portion of the constantly growing number of toxins. The usefulness of these research’ data and findings is debatable, despite the fact that they are instructive (Ceratti et al., 2021). This is because DNT research is frequently confusing and because it is impossible and unrealistic to investigate every single molecule. Therefore, the most obvious conclusion is that either the single or overall mode of action and cumulative effects of neurotoxicants cannot be definitively determined and acknowledged as ecologically sound. The growth of the neurological system depends on a cellular process called neuronal apoptosis, which is defined by programmed cell death (Guxens et al., 2018). For instance, post-mitotic neurons in a growing brain died as a result of synaptic links. Generally speaking, apoptosis is essential to preserving tissue integrity. Cell death has both benefits and drawbacks, much like any additional biological function. First, apoptosis induction makes it possible to remove potentially harmful cells and keep healthy cells out of the process. Uncontrolled apoptosis, on the other hand, can potentially result in tissue injury. In another situation, a build-up of injured cells may lead to low quantities of apoptosis (Argüelles et al., 2019). Necrosis and apoptosis are frequently confused.

Yet, during crucial stages of brain development, hazardous chemicals that can promote apoptosis may be able to trigger the programmed process. This may be detrimental since it may change mechanistic processes and impair cognition and behavior. Recent research by Zhu et al. (2021) found that neuronal apoptosis is still thought to have a role in the formation of neurodegenerative illnesses, despite the lack of evidence connecting PM2.5 exposure to neuronal damage brought on by cell death. A rate-limiting enzyme called S-adenosylmethionine decarboxylase 1 (AMD1) helps control cell death by reducing the frequency of this procedure. Additionally, it has been demonstrated that spermidine, a polyamine molecule that closely metabolizes AMD1, reduces neurodegeneration (Zhu et al., 2021).

Studies examining neuroapoptosis’s function in neurotoxicity have yet to fully elucidate its mechanism. On the other hand, the notion that programmed cell death raises the risk of certain neurodegenerative illnesses is a helpful place to start when trying to define bodily processes more precisely (Rosa et al., 2019). Numerous research suggest a possible link between neuroinflammation and apoptotic activation. Through the effective extraction of leukocyte DNA from cord blood and subsequent polymerase chain reaction analysis, telomere size. was successfully determined. It is interesting to note that the investigators also discovered that telomere length increased in pregnant women exposed to PM2.5 in the latter stages of pregnancy. Overall, since we know that decreased telomere length is a sign of biological aging, neuroinflammation may contribute to apoptosis (Singh et al., 2020). More precisely, telomere length is determined by DNA damage caused by oxidative stress, that is a component of systemic neuroinflammation. These findings imply that future neurotoxic research examining the connections among air pollution and neuronal death should focus on neuroinflammation.

## 7 Translational models and systemic mechanisms of developmental neurotoxicity across the lifespan

Because rat physiology and molecular signaling pathways are similar to those of humans, animal models continue to be essential to the study of DNT, providing mechanistic insights into exposure to neurotoxicants, Table 4. Experimental research shows that exposure to ultrafine particles, diesel engine exhaust, and PM2.5 during pregnancy and the early years of life disrupts synaptogenesis, changes behavior, and speeds up neurological pathology, involving the development of plaque resembling AD and phenotypes associated with ASD (Lane et al., 2025). The ongoing value of animal models is reinforced by the fact that, despite ethical concerns and the emergence of different strategies like stem and progenitor cell systems, these techniques are currently unable to capture intricate steps like neuronal maturation, glial differentiation, and higher cognitive outcomes. Experimental results are translated to children in megacities who are subjected to air contamination. *Via* gastrointestinal barrier disruption, circulatory systems, and olfactory movement, inhalation and ingestion pathways enable metals and particulate matter to enter the CNS (Ishihara et al., 2025). These mechanisms lead to neuroinflammation and early neuropathological features such tau hyperphosphorylation and amyloid buildup by triggering oxidative stress, microglial activation, and BBB damage. Due to their underdeveloped neurological and immunological systems, greater exposure behaviors, and developmental stages marked by fast myelination and neuronal expansion, children are more vulnerable (Wang H. et al., 2021).

**TABLE 4:** Systemic mechanisms, experimental models, and developmental neurotoxicity results related to environmental neurotoxins.

S.no	Model type	Exposure/Biological entry route	Experimental or population characteristics	Cellular molecular mechanisms reported	Neurological outcomes	Translational outcome	References
1.	Rodent animal models (general DNT research)	Neurotoxicant exposure in laboratory rodents	Genetically manipulable rodents; homologous physiological and anatomical facets	Hierarchical cascades and homologous disease mechanisms	Translational relevance to human neurodevelopment	Animal models remain indispensable despite ethical debates	Serafini et al. (2024)
2.	Alternative testing approaches	Stem and progenitor cell systems	Early-stage DNT testing platforms	Apoptosis, neural proliferation, neuronal migration studied	Limited coverage of neuronal maturation and glia differentiation	Cannot capture emotional or intellectual outcomes	Newell and Patisaul, (2025)
3.	Prenatal and early postnatal mouse exposure	Ultrafine particle exposure during late pregnancy and early life	Mouse laboratory investigation	Developmental neurotoxicity mechanisms	ASD-like behavioral characteristics	Early-life exposure parallels human developmental vulnerability	Shang et al. (2023)
4.	Transgenic mouse CNS studies	Diesel engine exhaust (DEE) exposure	Transgenic mice	Plaque formation acceleration; motor system impairment	AD-associated pathology	Long-term pollutant exposure linked with neurodegeneration	Hullmann et al. (2017)
5.	Rodent inhalation models	PM2.5 inhalation	Rodents exposed to particulate matter	Morphological and physiological alterations; synaptogenesis disruption	Memory deficits; spatial learning impairment; depressive behavior	Molecular disruption of neurodevelopmental processes	Liu X. et al. (2023)
6.	Young male F1 mouse models	PM2.5 exposure	Male F1 mice	Reduced IL-18 and vascular endothelial growth factor levels	Anxiety-related behaviors	Behavioral outcomes linked to inflammatory signaling	Nephew et al. (2020)
7.	Pediatric human population (megacities)	Inhalation and ingestion of PM	Children living in polluted urban environments	BBB impairment; microglial activation; systemic inflammation	Increased neurotoxicity vulnerability compared with adults	Immature immune and nervous systems increase susceptibility	D’Angiulli, (2018)
8.	Respiratory pathway exposure	PM inhalation through lungs and olfactory epithelium	Developing CNS	Translocation from lungs to bloodstream; inflammatory mediator circulation	CNS inflammation and neurological risk	Aerodynamic particle size determines distribution	Ishihara et al. (2025)
9.	Gastrointestinal pathway exposure	Ingestion of pollutants and heavy metals	Children and experimental animal observations	Tight junction disruption; mucosal barrier impairment; microbiota alteration	Intestinal inflammation; metabolic dysregulation	Gut–brain axis involvement in neurotoxicity	Hu et al. (2022)
10.	Microbiome disruption models	Heavy metal exposure (arsenic, cadmium, lead, mercury)	Gut microbiota studies	Altered metabolic profile; reduced bacterial growth; impaired detoxification	Progressive metabolic disease risk	Microbiome integrity modulates toxicant metabolism	Li X. et al. (2019)
11.	Respiratory system inflammation studies	Schoolchildren near pollution sources	Salamanca and other polluted regions	Endothelin-1 production; innate immune activation	Altered heart rhythm; vagal response impairment	Peripheral inflammation contributes to CNS effects	(Parenteau et al., 2022)
12.	Cytokine-mediated neuroinflammation	Systemic inflammatory mediator circulation	Pediatric populations	IL-1β, TNF, IL-6 signaling affecting brain vessels	Neurovascular compromise	Peripheral immune signals affect CNS integrity	Mallick et al. (2025)
13.	Neuropathological pediatric observations	UFPM accumulation *via* olfactory route	Children from MCMA	NVU damage; protein aggregation	Aβ plaques; tau hyperphosphorylation; α-synuclein accumulation	Early AD/PD hallmarks observed in youth	Calderón-Garcidueñas et al. (2024)
14.	Genetic susceptibility context	APOE allele carriers	Pediatric neuropathology cohorts	Interaction between genetic risk and PM exposure	Increased AD risk markers	Gene–environment interaction highlighted	Essers et al. (2023)
15.	Gut–brain axis mechanism	Intestinal inflammation and vagus nerve signaling	Neurodegenerative disease models	α-synuclein propagation from gut to brain	PD-related pathology progression	Enteric nervous system participates in DNT cascade	Yang R. et al. (2022)

Beyond the brain, pulmonary, gastrointestinal, and microbiome-related pathways are also involved in systemic inflammation. A bidirectional gut–brain axis that may increase the risk of PD and α-synuclein propagation is created by gut dysbiosis, heavy metal exposure, and cytokine signaling. The interplay of environmental variables, genetics, including APOE status, and socioeconomic determinants is highlighted by epidemiological studies that further link long-term particle exposure to cognitive impairments, dementia incidence, and behaviors associated with ASD (Park et al., 2025). All of these results point to neurotoxicants as causes of long-term neurodevelopmental and neurodegenerative risk, bolstering preventative measures that emphasize lowering exposure levels early in life.

## 8 Epigenetic changes induced by air pollution in neurodegenerative conditions

Even though the current understanding of epigenetics has become widely accepted for more than 10 years, it is still unclear exactly how epigenetic changes impact gene expression. Epigenetics offers a more comprehensive view of heredity than Neo-Darwinism, which rejects the inheritance of acquired traits and stresses random DNA mutations as the main force behind evolution (Danchin, 2022). It is now commonly acknowledged that genetic alterations can occur without reference to variances in DNA sequence. Following the initial exposure stops, environmental variables such as pollution, nutrition, and temperature can cause phenotypic changes that are passed down through the generations *via* epigenetic inheritance. Environmental influences can drastically change the epigenome, impacting processes like DNA methylation, modification of his and non-coding RNA production, even though they usually don’t change sequences of DNA. These epigenetic modifications impact mitotic stability, which in turn contributes to the onset of disease. and phenotypic diversity (Dickins, 2021).

As a non-Mendelian form of inheritance, environmental epigenetic transgenerational inheritance has significant consequences for biology of evolution, disease causation, and the inheritance of phenotypic features. Furthermore, it is important to consider genetics and environmental epigenetics as intricately linked molecular systems (Iqubal et al., 2020). According to research, long-term exposure to environmental contaminants, including industrial toxins, prior to and throughout pregnancy may play a major role in the development of neurodegenerative illnesses. Certain environmental agents, such as pesticides, nanoparticles, polychlorinated biphenyls, bisphenol A, and heavy metals (lead, mercury, and arsenic), have been linked to the occurrence of key neuropathological characteristics of neurodegenerative diseases, such as the build-up of neurofibrillary tangles and the emergence of amyloid plaque (Yu, et al., 2021b). Environmental pollutants can alter mRNA stability, protein synthesis, and epigenetic alterations by interfering with the complex regulatory processes that control gene activity. In particular, changes in histone protein alterations, non-coding RNA expression, and DNA methylation patterns can all be biomarkers of encountering neurotoxic pollutants in the environment (Gu C. et al., 2020).

### 8.1 DNA methylation

AD is a form of dementia that mostly affects the hippocampus and cortex, two areas that are essential for learning and memory. The buildup of aberrant proteins, such as clusters of amyloid beta protein (Aβ) outside of neurons (senile plaques) and tangled Tau proteins inside neurons (neurofibrillary tangles), is a defining feature of AD. The abnormal patterns of gene expression seen in the course of AD are thought to be caused in part by DNA methylation, an action that controls gene transcription (Balmik and Chinnathambi, 2021). Research has indicated that AD patients are more likely to have reduced level of DNA methylation in genes linked to Aβ production, such as PS1 and APP. Furthermore, there is a connection involving Tau protein neurofibrillary tangles in AD patients and decreased DNA methylation in the BACE1 promoter, which is connected to Aβ deposition (Wei et al., 2021). PM2.5-triggered inflammation and oxidative stress may result in epigenetic alterations and instability of the genome, such as altered DNA methylation at CpG sites linked to neuroinflammation genes like SORBS2, PDE11A, and GABBR1 (Jiang et al., 2025). Research indicates that exposure to PM2.5 may cause variations in DNA methylation similar to those found in AD, such as BACE1, APP, PS1, and APOE. DNA methylation patterns are also influenced by environmental variables other than air pollution. Promoter methylation changes, CpG island hypo- and hypermethylation, and altered methylation of genes controlling neurological growth and dopaminergic transmission have all been associated with exposure to heavy metals, polychlorinated biphenyls, pesticides, and bisphenol A (Liu M. et al., 2023). DNA methylation is found in the CpG-2–4 regions of the SHANK3 gene in postmortem brain tissue from people with autism spectrum disorder (Montanari et al., 2022). DNA methylation is found in the CpG-2–4 regions of the SHANK3 gene in autopsy brain tissue from people with ASD. According to genome-wide association research, exposure to ambient PCBs and polycyclic aromatic hydrocarbons changes the DNA methylation of genes including Crebbp, Hdac5, Mecp2, and Dnmt3a, which may be a factor in behaviors resembling ADHD (Marques et al., 2025).

### 8.2 Histone modification

Histone acetyltransferases and histone deacetylases control the dynamic epigenetic process of histone acetylation, which is essential for brain activity. Histone alterations have been connected during research to neurodegenerative illnesses like AD. Exposure to environmental pollutants can seriously interfere with this process. While reduced H3K27 acetylation in Alzheimer’s mouse brains impacts synaptic plasticity genes, H3K9 acetylation is associated with stem cell formation and neural differentiation (Santana et al., 2022). Memory and synaptic function are enhanced by modifying histone acetylation through the use of histone acetyltransferase stimulants or antagonists of histone deacetylase.

By changing histone acetylation and methylation trends, environmental chemicals that affect brain function go beyond air pollution. These include pesticides like dieldrin and paraquat, endocrine disruptors like bisphenol A, and heavy metals like lead, arsenic, and nickel. Changes in Nos1, Sez6l, Kcna6, and Pml gene expression are correlated with decreased H3K9 acetylation (Zhou et al., 2021). Dieldrin injection raises histone acetylation in the striatal and substantia nigra areas, whereas nickel exposure causes histone hypoacetylation. Because paraquat reduces HDAC action, it causes histone H3 acetylation. Chronic exposure to PM2.5 changes histone methylation patterns in prefrontal white matter nuclei, resulting in increased amounts of γ-H2A.X and lower levels of H3K9me2/me3, which causes DNA damage, genomic instability, and aberrant gene transcription (Calderón-Garcidueñas et al., 2020).

### 8.3 Non-coding RNAs

The production of non-coding RNAs, notably microRNAs, is dramatically changed by exposition to ambient PM, ultrafine particles, diesel exhaust particles, carbon black nanoparticles, pesticides, and neurotoxicants like MPTP. These tiny non-coding RNAs control the production of proteins and adjust how cells react to external stimuli. Non-coding RNA expression controlling neuroprotective metallothioneins MT1A and MT1F, which are linked to the advancement of neurodegenerative diseases, is changed by UFP exposure (Cheng et al., 2025). Cognitive impairment and neurodegenerative illnesses like AD have been linked to changes in microRNA profiles or impairments in Dicer or DGCR8. Exposure to PM2.5 inhibits miR-574-5p, which raises BACE1 expression and impairs synaptic function (Ku et al., 2017). Rotenone and paraquat are examples of pesticides that change the expression of miRNAs, such as miR-384-5p, miR-195, and miR-200a, which leads to the death of brain progenitor cells and disturbed differentiation. In models of PD, MPTP controls miR-494, miR-7, and miR-124, which affects apoptosis, autophagy, and oxidative stress cascades (Segaran et al., 2020). According to data from the ENVIRONAGE study, exposure to environmental pollutants during pregnancy impacts epigenetic variations such as DNA methylation, histone modifications, and non-coding RNA expression. These changes result in modified newborn phenotypes and a higher risk of NDD in later life (Cox et al., 2019).

## 9 Correlation between environmental neurotoxicant-driven mechanisms and epigenetic alterations

As was previously mentioned, environmental neurotoxins like PM, gaseous pollutants, pesticides, and heavy metals interact with one another to cause oxidative stress and neuroinflammation, which are upstream signals for modifications in the maintenance of DNA methylation, histone remodeling/reprogramming, and misregulation of non-coding RNA (Stoccoro and Coppedè, 2024). These interconnected pathways show how exposure to the environment is converted into persistent molecular states that affect synaptic plasticity, neuronal survival, and disease risk over the course of a person’s lifetime.

### 9.1 Neurotoxicant exposure pathway and DNA methylation

The documented biochemical pathways of particulate matter-induced brain injury and the development of neurodegenerative markers are closely linked to the alterations in DNA methylation discussed previously. The aforementioned sections addressed oxidative damage, NFκB activation, and inflammatory cytokine signaling following exposure to PM2.5; these alterations are also connected to altered methylation of other genes (APP, BACE1, PS1, and APOE), thereby bolstering the connection between environmental exposure and amyloidogenesis (Thiankhaw et al., 2022). It is suggested that DNA methylation is a downstream molecular imprint of cumulative exposome interactions because metals, pesticides, and industrial neurotoxicants that are mentioned in the brain development neurotoxicity also contribute to methylation CpG island changes, neuronal differentiation divergences, and—most importantly—alterations in neurotransmission. The link among prenatal exposure and gene patterns of methylation and neurodevelopmental abnormalities linked to ASD further supports the incorporation of epigenetic consequences into a life-course susceptibility that was highlighted in earlier lifespan models (LaSalle, 2025).

### 9.2 Histone changes associated with oxidative stress, neuroinflammation, and apoptosis

The processes of oxidative stress and mitochondrial malfunction discussed in the section on inflammation and neuronal death are correlated with changes in histone acetylation and methylation. Reduced H3K9me2/me3 and elevated γ-H2A. X expression are consistent with PM exposure, which has been shown to cause nitrooxidative stress and DNA damage. These findings directly link chromatin remodeling with genomic instability (Calderón-Garcidueñas et al., 2020). Histone acetylation levels, which control the patterns of expression for genes involved in synaptic plasticity, are impacted by neurotoxicants like paraquat, dieldrin, and heavy metals, which are also listed in the environmental neurotoxicant tables. Prior observations of impaired learning, behavioral alterations, and accelerated AD-like disease in these models can be explained by these epigenetic disruptions. Chromatin remodeling is the point of convergence for environmental stressors and neuronal cell death cascades, as demonstrated once again by the interaction between histone and apoptotic pathways (Janowski et al., 2021).

### 9.3 Non-coding RNAs as integrators of environmental exposure and neurodegenerative signaling

The changes in ncRNAs align with the previously described pathways for translational models and particulate matter-induced neurodegenerative indicators. MicroRNAs, such as miR-574-5p, which has been linked to PM2.5 exposure in the past, show how environmental stimuli alter post-transcriptional regulation to promote amyloidogenic pathways (Mi et al., 2025). In experimental scenarios involving MPTP, rotenone, and diesel exhaust, where the miRNA profile is altered due to oxidative stress and autophagy failure, additional mechanistic connections between environmental toxicity and epigenetic control can be discovered (Angelopoulou et al., 2022). Furthermore, the growing evidence from developmental exposure models that shows alterations in newborn epigenetic readouts highlights the function of non-coding RNAs as molecular bridges that connect early environmental exposures to later neurodegenerative risk.

## 10 Limitation

Despite the depth of the mechanical discussion offered, the research raises a number of cautions. The fact that exposure assessments vary from study to study, particularly with regard to PM composition, exposure duration, and complicated pollutant mixes, is one of the primary problems. Even though they are mechanistically instructive, a sizable portion of experimental data is derived from animal models or *in vitro* systems that may not accurately reflect the intricacy of human neurodevelopment and long-term environmental exposure patterns. Broader translational gaps still exist, especially when it comes to distinguishing dose-response effects and establishing thresholds that are clinically relevant in real-world exposure scenarios (Mangrulkar et al., 2024). Since environmentally relevant exposure frequently happens in combination, separating out epigenetic modifications that are directly linked to certain contaminants presents another difficulty. Furthermore, it is still unknown to what degree these signatures accurately represent long-term changes in the human brain because most epigenetic correlations are found in peripheral tissues or in short-term observational studies. Another area of uncertainty is the methodological diversity among epigenetic research. It is challenging to develop unifying biomarkers and studies are less comparable when methylation arrays, sequencing platforms, and analytical pipelines are heterogeneous. Furthermore, there are few longitudinal human studies that link early life epigenetic alterations and clinically diagnosed neurodegenerative endpoints, despite the manuscript’s strong molecular connections (Mitra et al., 2025). These drawbacks emphasize how crucial it is for future research to use harmonized exposure measurements in conjunction with integrated multi-omics.

## 11 Future direction: Multi-omics approaches and cell-type-specific epigenetic regulation

Despite tremendous advancements, the bulk of the epigenomic research on NDDs has relied on bulk tissue investigations that temporize cell and home-type devices, limiting our ability to identify the mechanisms behind the alteration of specific neurons and glial populations. The solution to this challenge is provided by the development of single-cell technology. Transcriptional states, available regulatory elements, and epigenetic fingerprints of thousands of individual cells can be mapped using single-cell RNA sequencing (scRNA-seq), single-cell chromatin accessibility characterization (scATAC-seq), and single-cell DNA methylation analysis (Preissl et al., 2023). Progenitors, excitatory neurons, inhibitory neurons, and glial precursors—all of which go through very divergent lineage programs related to NDDs—are among the diverse brain populations that can be studied with the help of these modalities. The ability to incorporate these molecular levels and reconstruct vast regulatory ecosystems is made possible by integrative multi-omics techniques. Transcriptomic, epigenomic, and chromatin-accessibility data can be harmonized into single cell-state representations using canonical correlation analysis (CCA), manifold alignment, mutual nearest neighbor (MNN), and weighted nearest-neighbor integration (Alshardan et al., 2026). These methods may be employed to find convergent pathways that are not visible with single modalities in human fetal brain tissue, animal developmental periods, and patient-generated neural progenitor networks.

Cellular programming and epigenetics explain how different cell types are produced from identical genetic material through controlled gene expression. DNA methylation, histone changes, and non-coding RNAs govern this process, which establishes cellular identity by controlling gene activation and silencing. Throughout development, epigenetic programming is dynamic and includes cell-specific changes that direct neuronal differentiation as well as genome-wide demethylation followed by remethylation (Saleh et al., 2026). Environmental variables also have an impact on these processes *via* epigenetic reprogramming. Through a pathway of psychological challenges and inflammation, early life stress (ELS) links developmental programming to resulting neurodegenerative risk by contributing to long-lasting epigenetic impacts on circuits governing brain growth, immune systems, and behavior.

In order to shed light on the factors underlying these NDDs at the tissue, cellular, and network levels, initiatives continue to advance from genetics alone and now condition their work by incorporating genomic, transcriptomic, proteomic, epigenetic, and metabolomic data along with neuroimaging information (Chen et al., 2021). System-level studies of molecular interaction networks that result in complex polygenic illnesses are made possible by high-throughput omics integration. These networks emphasize interconnected regulatory landscapes rather than linear paths and involve, but are not limited to, protein–protein interactions, gene co-expression networks, gene regulatory networks, and chromatin interaction networks (Cocoş and Popescu, 2024). Molecular signatures may now be separated according to the kind of cell thanks to developments in single-cell omic technologies. Tens of thousands of transcriptome data can be obtained at once using single-cell RNA sequencing (scRNA-seq) and single-nucleus RNA sequencing (snRNA-seq), which makes it possible to illustrate cell-type-dependent regulatory processes in neurodegenerative diseases. Divergent expression patterns between neuronal and glial populations are characteristic of cellular heterogeneity in neurodegeneration, as demonstrated by applications in AD, PD, ALS, FTD, DLB, and HD (Martirosyan et al., 2024; Goddard et al., 2024). DEG analysis has been used to build tissue- and cell-type-specific regulatory networks that show distinct expression patterns in astrocytes, microglia, oligodendrocytes, and several neuronal subtypes under illness settings. The importance of cell-type-specific epigenetic control in the genesis of disease is further demonstrated by these findings (Mathys et al., 2019).

Additionally, inter-layer integration of genetic and molecular variations is made possible by multimodal frameworks like xQTLs, which also include eQTLs, pQTLs, epigenetic, and metabolomic QTLs (Pan S. et al., 2024). Facilitating the integration of xQTL datasets improves the interpretation of genome-wide association studies and helps prioritize genes connected to functional neurodegenerative traits. To find genes influencing disease through genetically regulated expression, transcriptome-wide association studies (TWAS) further combine GWAS and eQTL data (Sun et al., 2021). These methods show the role of both central and peripheral systems in neurodegenerative pathology by revealing tissue-specific and cross-tissue regulation mechanisms. Together, these developments indicate that future research should focus on single-cell resolution techniques and multi-omics integration in order to depict heterogeneous regulatory landscapes, identify cell-type-specific epigenetic mechanisms, and identify therapeutic targets in NDDs.

## 12 Discussion

Since environmental exposures should be viewed as long-term regulators rather than just harmful episodes, the body of evidence presented in this research supports the multifaceted view of neurodegeneration. Instead of being separate toxic exposures, current findings show that environmental neurotoxicants actively modulate neurodegenerative disease through interrelated biological processes. Particulate matter, heavy metals, gaseous pollutants, and synthetic toxicants all contribute to oxidative stress, mitochondrial dysfunction, and neuroinflammatory signaling, which in turn converge upon epigenetic dysregulation that advances neuronal injury. The fact that oxidative stress and neuroinflammation function as upstream routes of downstream disease processes is a crucial molecular understanding. Environmental contaminants produce reactive oxygen and nitrogen species, which trigger NF-κB → cytokine release signaling pathways to sustain conditions linked to chronic inflammation. Because persistent inflammation exacerbates oxidative imbalance, this creates a positive feedback loop of cellular damage. Concurrently, mitochondrial malfunction with reduced ATP generation, compromised membrane integrity, and mtDNA damage is identified as a crucial element linking environmental stress to disrupted neuronal energetics, which is followed by heightened susceptibility to apoptosis.

Many known upstream disruptions, whether constitutional or environmental, converge on disease-relevant molecular pathways, which in turn govern processes like amyloidogenesis and synaptic dysfunction. It has been shown that exposure to pollutants increases BACE1 through NF-κB activation, which leads to the synthesis of amyloid-β. On the other hand, microRNA dysregulation, such as a decrease in miR-574-5p, may worsen synaptic impairment and cognitive decline. These results show that environmental exposures have a direct effect on canonical neurodegenerative pathways and are more likely to act through the same pathways that underlie these disorders rather than acting as risk modifiers in and of themselves. This review notably emphasizes the importance of epigenetic pathways as integrative mediators. The heritable changes in DNA methylation, histone modifications, and non-coding RNA expression are responsible for the persistent transmission of epigenetic signals in gene regulation from brief environmental exposures to progeny. The long-lasting consequences of environmental stressors may be explained by these epigenetic changes that affect genes controlling neuroinflammation, synaptic plasticity, and neuronal survival. In this case, epigenetic dysregulation is a crucial factor in the pathophysiology of the disease rather than only a result.

The results provide credence to a systemic model of neurotoxicity in which environmental chemicals enter the brain through multiple channels, such as the gastrointestinal, circulatory, and olfactory systems, and cause significant biological disruption outside of the central nervous system. These mechanisms have a lot in common with what is described in severe mental diseases, such as major depression and schizophrenia, when considered in the context of historical literature. Environmental pollutants are linked to oxidative stress, neuroinflammation, mitochondrial dysfunction, and epigenetic modifications in both domains. This suggests that environmental exposures may operate along a common mechanistic continuum, with time, genetic vulnerability, and cumulative exposure load determining divergence. All of these findings point to an exposome-epigenome paradigm that explains how environmental neurotoxicants affect the risk of neurodegenerative illness by interacting with gene-regulatory networks and biological resistance throughout the lifetime.

## 13 Conclusion

According to the reviewed body of research, environmental neurotoxicants modify the risk of neurodegenerative disease by means of interrelated processes that include oxidative stress, inflammation, mitochondrial malfunction, and epigenetic rewiring. The main molecular mechanisms *via* which environmental exposure penetrates the skin to cause long-lasting alterations in neural gene expression and cellular resilience include modifications in DNA methylation, histone modification, and non-coding RNA expression. Rather than operating independently, these epigenetic changes convert PM exposure and developmental neurotoxicity signals into apoptotic pathways and systemic inflammatory responses, which come together to provide a cohesive framework linking environmental insults to neurological impacts. In the development and progression of neurodegeneration, the review highlights the dynamic interplay between the genome and exposome, where environmental variables influence the susceptibility to illness throughout life. Early exposure is identified as a critical period during which an individual’s epigenetic programming may predispose them to later neurodegenerative pathology. This finding has implications for environmental factor-based prevention measures. Environmentally produced epigenetic fingerprints may be useful as early illness biomarkers and therapeutic intervention targets, according to recent advancements in epigenomic profiling and translational models.

Cohort studies, exposure assessment harmonization, and collaborative initiatives toward integrative genomics/epigenomics/environmental sciences analysis will be necessary for future advancements. Cohort studies, exposure assessment harmonization, and collaborative initiatives toward integrative genomics/epigenomics/environmental sciences analysis will be necessary for future advancements. A paradigm where risk is modifiable through improved environmental knowledge, public health initiatives, and precision interventions aimed at protecting neurological health is supported by placing knowledge of neurodegenerative disease within the framework of an environmental epigenetic model.

## Data Availability

The original contributions presented in the study are included in the article/Supplementary Material, further inquiries can be directed to the corresponding author.
