# Supplementary material for: Environmental epigenetic modifiers in neurodegenerative diseases: a systematic review of epigenomic mechanisms and therapeutic targets
Source: Front Epigenet Epigenom. 2026 May 28;4:1833533. doi: 10.3389/freae.2026.1833533 (PMC13632485; doi:10.3389/freae.2026.1833533)
Supplement: Supplementary file 1 [file DataSheet1.pdf]

First Author	Year	Study Design	Population / Model	Pollutant(s)	Epigenetic Mechanism	Key Findings / Conclusions
Adekomi	2024	Experimental (in vivo)	Juvenile BALB/c mice	Arsenic, Vanadium	Not reported	Co-exposure caused synergistic cerebellar neurotoxicity, increased apoptosis, inflammation, oxidative stress, and locomotor deficits.
Angelopoulou	2021	Narrative Review	Human & preclinical studies	Heavy metals, pesticides, lifestyle factors	DNA methylation, histone modification, ncRNAs	Environmental factors contribute to Parkinson's disease via epigenetic regulation of gene expression.
Cox	2019	Birth cohort study	427 mother–newborn pairs	PM2.5, PM10, NO2, BC	Not reported	Prenatal air pollution exposure associated with increased neonatal blood pressure.
Calderón-Garcidueñas	2018	Observational (post-mortem)	Humans (≤40 years, Mexico City)	PM2.5, combustion-derived nanoparticles	Not reported	Early AD and PD pathology (tau, Lewy neurites) observed in young individuals exposed to air pollution.
Calderón-Garcidueñas	2020	Human + animal study	Humans & mice	Particulate matter (CFDNPs)	Histone modification (↓H3K9me2/3), DNA damage (γ-H2AX)	Air pollution reduces repressive histone marks and increases DNA damage, promoting AD pathology.
Calderón-Garcidueñas	2021	Review	Human & experimental evidence	PM2.5, UFPs,	Not specified (mechanistic discussion)	Particle pollution induces oxidative stress, neuroinflammati

				nanoparticles		on, and increases risk of neuropsychiatric disorders.
Calderón-Garcidueñas	2020	Observational (autopsy)	Humans (children & young adults)	UFPM, industrial nanoparticles (metals)	Not reported	Early-life exposure linked to AD, PD, ALS neuropathology and widespread brain damage.
Essers	2022	Cohort study	1186 children	PMcoarse, PAHs	Not directly epigenetic (genetic modifiers: APOE, PRS)	Air pollution affects brain morphology; genetic factors modify susceptibility.
Gu	2020	Experimental (C. elegans)	C. elegans (F0–F6 generations)	Arsenite	Histone modification (↑H3K4me2 via ↓spr-5)	Arsenite induces transgenerational metabolic effects via histone methylation changes.
Gui	2023	Experimental (in vivo)	Juvenile rats	Particulate matter	Not reported	PM exposure impaired learning and memory via synaptic dysfunction and gene alterations.
Hu	2023	Experimental (in vivo)	Mice	Lead (Pb)	Not reported	Lead worsens metabolic dysfunction via gut microbiome disruption and inflammation.
Hullmann	2017	Experimental (in vivo)	5XFAD mice	Diesel exhaust	Not reported	Diesel exhaust accelerates amyloid plaque formation and motor deficits.
Ibarra-Gutiérrez	2023	Review	Experimental models	Rotenone	Not primarily epigenetic	Rotenone induces PD-like pathology via mitochondrial dysfunction and α-synuclein aggregation.

Ishihara	2025	Review	Experimental & human studies	PM2.5 components	Not specified	PM2.5 components enter brain and induce neurotoxicity via multiple mechanisms.
Liu	2020	Review	Human & animal studies	Lead	DNA methylation, histone modification	Lead exposure alters epigenetic landscape contributing to neurodevelopmental disorders.
Liu (Xin-qi)	2023	Review	Human & animal studies	PM2.5	DNA hypomethylation	PM2.5 induces epigenetic changes contributing to CNS damage and neurodegeneration.
Ku	2017	Experimental (in vivo)	Mice	PM2.5	miRNA regulation (↓miR-574-5p via NF-κB)	PM2.5 induces cognitive impairment via epigenetic regulation of BACE1.
Li (Ben)	2017	Experimental (in vitro/in vivo)	Neurons & brain slices	PM2.5	Indirect (NF-κB signaling)	PM2.5 induces synaptic dysfunction via ROS–NF-κB–COX-2 pathway.
Li (Xuanji)	2019	Experimental (in vivo)	Mice	Arsenic, Cadmium	Not reported	Heavy metals alter gut microbiome and metabolome linked to metabolic disease.
Liu (Mengchen)	2022	Review	Human, animal, cell models	Lead	DNA methylation, histone modification	Lead-induced epigenetic alterations linked to cognitive and neurodegenerative disorders.
Nephew	2020	Experimental (in vivo)	Rats	Traffic-related PM	Not reported	Developmental PM exposure causes anxiety,

						cognitive deficits, and neural damage.
Zhou	2021	Experimental (in vivo/in vitro)	Mice & neurons	Nickel	Histone acetylation (↓H3K9ac)	Nickel-induced neurotoxicity mediated by histone hypoacetylation and gene repression.
Zhu	2021	Experimental (in vivo/in vitro)	Mice & neurons	PM2.5	Not directly epigenetic (metabolic pathway)	PM2.5 induces neuronal apoptosis via AMD1/spermidine pathway.
